# Switching intention to crypto-currency market: Factors predisposing some individuals to risky investment

**DOI:** 10.1371/journal.pone.0234155

**Published:** 2020-06-04

**Authors:** Wei Sun, Alisher Tohirovich Dedahanov, Ho Young Shin, Ki Su Kim

**Affiliations:** 1 School of Economic, Anyang Normal University, Anyang city, Henan Province, China; 2 School of Business, Yeungnam University, Gyeongsan, Korea; University of Almeria, SPAIN

## Abstract

We investigate factors affecting individual investors’ switching intention from traditional financial market to crypto-currency financial market. By sampling factors of individual investors related with crypto-currency (CC), the study applies structural equation modeling method (SEM) to investigate their effects on switching intention by integrating PPM and Reinforcement Sensitivity theories (RST) to form a pulling, pushing and mooring effects model. The investigation indicates that crypto-currency market can be regarded as a kind of beneficial supplement of tradition investment market for those individual investors who are with high innovativeness, reward sensitivity, knowledge and perceived risk. This study proves that the individual investors are not only attracted by significant expected return from crypto-currency but also relevant knowledge and risks disclosed by crypto-currency market regulators and distributors. The findings reinforce major roles for both market regulators and individual investors in considering and providing insights for future policy, management and investigations.

## 1. Introduction

Crypto-currencies (CC) are rapidly gaining more and more interest as a technology that is potentially ground breaking and disruptive for the whole payments industry on a global scale. The two largest economic entities, the United States and China have declared to develop their own crypto-currencies. Crypto-currency is a speculative financial asset with expected return, risk and volatility as well as ordinary currencies. Although crypto-currency has been gained great attention in financial markets, little attention has ever been paid in individual investors’ perspectives. Furthermore, current researches on the crypto-currencies mostly focus on the expected return, volatility and risk, ignoring the behavior perspectives in crypto-currencies among individual investors [1].

Usually, in order to pursue capital maintenance, more winning or permanent income, most individual investors have to give their own fortune to private bank, trust, and insurance companies etc. to get limited benefit. In fact, since trade barriers between great powers are pulling out of global economy recession, it is getting harder for individual investors to find better investment opportunities until the crypto-currency appears. Crypto-currencies (CC hereafter), such as Bitcoin, Ripple, Bitcoin Cash, and Litecoin etc., have boldly moved in focus of attention in recent years. The related literature on CC is much more popular now, including Folkinshteyn & Lennon (2016) [2], Francisco & Swanson (2018) [3], Gunawan and Novendra (2017) [4] and Presthusa & O'Malley (2017) [5]. As for individual investment behavior research, there are seldom previous articles have ever focused on CC as a part of individual investors’ portfolio. Although individuals’ unfamiliarity to CC may lead to individuals take it for granted that CC is a kind of risky choice of their portfolio, some adventurous or innovative ones have put CC into their portfolios. Some crypto-currency companies have also actively used sophisticated marketing, social network service and mass media as publicity tools to strengthen promotion [17], which lead to the increasing global influence of virtual currencies. Therefore, it is necessary to apply proper theories about the investment behavior to find what factors affect those behaviors and give some instructions on how to deal with CC for individual investors.

Push-pull-mooring (PPM hereafter) theory includes three important factors. The first one is pushing-effect, which indicates the factor that drives individual investors away from traditional financial market to CC investment market in describing the possibility of low return and high loss [6]. Pull effect is the second one, which attracts individual investors to adopt CC as a useful investment tool. The last but not the least one is mooring effect, which indicates those factors that may constrain individual investors’ switching intention behavior [7]. Thus, this research is designed to examine mooring, pull, and push’s related effects on intention of switching empirically. It also examines how the mooring variable moderately influences the push-pull factors and individual investors’ switching intentions’ relationships, which can help market regulators managerial comprehend individual investors’ intention behaviors and enable them to improve social interactions in attracting more individual investors to lead to better investment return [8].

This study tries to explore how crypto-currencies affect the individual investors’ switching intention of investment behaviors to CC financial market with different characters of adoption progress. Also, since only limited previous researches apply information system theories of individual financial investment science, the most pioneer work of this investigation is to integrate two popular theories (PPM & RST) to investigate roles of crypto-currencies in individual investment behavior field. Specially, this research firstly combines behavioral psychology factors with information management science to investigate the individual investors’ switching intention behavior, which provides empirical evidence to fill the gap.

Previous articles of the relationship between switching intention and PPM theory are plenty, including online shopping behavior [8], security adoption [9], as well as many other research fields [6,10]. In this research, push factors are used to describe large traditional financial market’s disadvantage, while pull factors are used to indicate individual investors’ advantages in CC. As for mooring factor, it is used to act as a moderating constraint to moderate relations of individual investors’ switching intention.

### 1.1 Literature review

We have reviewed crypto-currency researches at different levels of analysis. Most previous researches have applied different theories equally well at financial market, management and organizational levels of analysis, and in those case there were sufficient data and cases to prove the importance of crypto-currency. What still remains to be discovered is if the PPM theory and RST are appropriated for individual investment behavior. Some of the old dimensions in those previous researches may no longer be relevant or need to be measured differently for individual investment progress. After considering many different previous researches about crypto-currency under a variety of conditions, we used PPM & RST in evaluating individuals’ investment behavior in crypto-currency. Table 1 shows the summary of some leading crypto-currency researches using varieties of methodologies in investment or portfolio management content, which shows that crypto-currency has revealed in many ways in financial research fields.

**Table 1:** Previous crypto-currency researches in financial fields.

Authors	Research Issues	Methodology
Cheah & Fry (2015) [11]	Investigation fundamental value of crypto-currency	Undertaking economic & econometric modeling
Dyhrberg (2016) [12]	Comparing crypto-currency with gold and dollar	A GARCH volatility analysis
Fry & Cheah (2016) [13]	Negative bubbles & shocks in crypto-currency markets	Modeling for financial bubbles and crashes
Pieters & Vivanco(2017) [14]	Regulations across crypto-currency markets	Examining differences Across Bitcoin markets
Bouri et al. (2017) [15]	Crypto-currency’s hedge functions for world stock	Dynamic conditional 2 correlation modeling
Brauneis & Mestel (2018) [16]	Price discovery of Crypto-currencies	Non-parametric test for market efficiency
Koutmos (2018) [17]	crypto-currency returns and transaction activity	Time series plots of logarithmic levels
Juhász et al. (2018) [18]	Identifying Bitcoin users	Bayesian approach
Samet Gunay (2019) [19]	Public Information Arrivals on Crypto-currency Market	Kapetanios test, Markov analysis, Maki analysis,
Nunez et al. (2019) [20]	Bitcoin during explosive behavior periods	Normal inverse Gaussian distribution
Dimitrova et al. (2019) [21]	Bitcoin market (in)efficiency	FD4 approach & FD algorithm etc.
Lahmiri & Bekiros (2019) [22]	Chaotic dynamics of crypto-currency series	Multi-step nonlinear decomposition approach
Kumar & Anandarao (2019) [23]	Volatility spillover in crypto-currency markets	GARC Hand wavelet analysis
Tran & Leirvik (2019) [24]	Efficiency in crypto-currencies markets	AMIM measure

## 2. Hypotheses model

### 2.1 Perceived risk and switching intention

Risk factor has been applied in variety research fields to explain different uncertainties, including cultural difference, economic, purchase behavior, personal privacy, situation and functional risk [25–30]. As more and more studies indicate that push effects usually can be representing a certain degree of negative effect, such as loss in money or time, privacy stealing, and price fluctuation that may lead to unsatisfying payback and loss of principal, this research try to use perceived risk to explain how CC could be a new investment choice. On one hand, since institutional investment dominates traditional financial markets, it is risky and difficult for individual investors to conveniently find suitable investment products in traditional financial market, and it may cost too much to get satisfying expected return as well. On the other hand, because the crypto-currency market currently consists of thousands of crypto-currencies, and each of them has its peculiarities and different levels of risk or profit/loss, individual investors can focus on certain specific crypto-currencies based on different risk tolerance and profit expectations, without having to invest in all varieties. Therefore, perceived risk can be used to describe the potential loss in pursuit of wealth or return on traditional financial market in order to drive individual investors away from traditional financial market. Perceived risk could make individual investors unsatisfied with traditional financial market. Some individual investors prefer to invest in CC to get more profits or seek a fair competition circumstances, because some individual investors may think that traditional financial markets have been monopolized by investment institutions. No matter how easy to invest in traditional financial market, due to yearning for new investment opportunities, most of the individual investors may intend to try in CC market. Additionally, although the most traditional financial tools are difficult to be put into portfolio, which may reduce individual investors’ enthusiasm, it may not happen in CC because there are so many brands and trading platforms. Individual investors can find plenty of information before making their choice. By the way, since it is hard to compare the perceived risk of investing in traditional financial market with others, people may prefer to have a try in new market for better return. Both Stone & Gronhaug (2007) [26] and Tuu et al. (2011) [31] have indicated that risk factors are related with users’ intention or adoption. Therefore, this research would like to propose that:

H1: Perceived risk of CC in traditional financial market positively affects the switching intention to CC.

### 2.2 Reinforcement sensitivity theory, reward sensitivity and knowledge

RST is an outstanding and helpful explanation about human behavior in motivation, learning and emotion [32,33]. Corr (2004) [34] used reward sensitivity to indicate that humans are sensitive and active in their daily life with different motivations, which may indicate intention for thoughts or behaviors that offer happiness. Avila et al. (2008) [35] indicated that humans who are holding high reward sensitivity may intend to possess high positive attitude to be after reward, as well as Avila et al. (2003) [36] indicated that humans are different when they have to deal with fast change situations, which means the higher reward sensitivity humans possess the higher performance they have. Since the article tries to retrieve how CC attracts individual investors to adopt it, we introduce reward sensitivity as an individual investors’ switching intention about what individual investors’ reward anticipation is during the before and after investment behavior.

RST is used in factors of pulling in order to increase individual investors’ switching to take CC as an explanation of traditional financial market. Meanwhile, it can empower switching behavior based some advantages about CC indicating that individual investors are going to achieve more returns or rewards by having positive thoughts with inclining to choosing CC as an investment target. Therefore, it can enhance switching to adopt CC to find investment opportunity. Because of individual investors’ higher intention for reward incentives, individual investors with stronger intention for better reward and better expected return to investment may take CC as a better option. Thus:

H2: Reward sensitivity about CC positively affects switching intention to CC.

Both Rindfleisch & Moorman (2001) [37] and Chang (2017) [38] proves that knowledge includes technics, specifications, data, features, interpersonal relationship, components and tools that individual has to possess in dealing with certain works or tasks. In this research, the knowledge could be taken on as some perception about what individual investors have about CC as an investing option and related techniques, attributes and underlying features. Previous researches have proved that knowledge played an important role in different situation on adoptions, including adoptions of new technology and product [9,39,40]. No individual investors would like to try in CC markets until they feel they have enough knowledge about new things [41,42]. If individual investors get enough information, skills, instruction, positive and negative news and are ware of advantages and benefits of CC, they may prepare themselves for switching to CC investment with more confidence. Thus,

H3: Knowledge about CC positively affects switching intention to CC.

### 2.3 Mooring effect (personal innovativeness)

Factors of mooring may increase individual investors’ switching behavior to try in CC to moderate relations among the adoption progress. Rogers (2005) [43] defines personal innovativeness as the degree that individuals are relatively earlier to adopt new ideas than other social system members. In CC investment field, personal innovativeness indicates individual investors’ intention to regard CC as an optional investment tool. Personal innovativeness has been proved to be an effective factor in different research areas. Hwang (2014) [44] identifies the personal innovativeness’s role in ERP system adoption. Lee et al. (2007) [45] proves personal innovativeness’s moderating effects on online travel shopping behavior. Lin & Filieri (2015) [46] investigate personal innovativeness’s role in airline passengers’ continuance intention. Individual investors comparatively possess more intention to adopt new technologies because they are rarely restricted by rules, regulations and ordinations as institutional investors are. Specially, since the personal innovation among individual investors is based on flow of investment ideas, CC knowledge and trading technics, the individual investors’ co-works in social network groups and off-line clubs can thus be looked on as innovation progress to reduce switching intention’s complexity and uncertainty. The collaboration with individual investors across different backgrounds leads to more creativity by the innovation progress. Moreover, individual investors can hold more positive aspect with regard to innovation features of same optional conditions through the combination of creative technology, highly expected reward and relatively low perceived risk. When individual investors possess higher personal innovativeness, not only their switching intention to CC will increase, but also the acceptance of the related pushing and pulling factors will increase higher. In other words, when individual investors would like to be more innovative, the pushing and pulling variables will be more important to the development of switching intention to CC. Therefore:

H4: Personal innovativeness positively affects switching intention to CC.
H5: The relationship between perceived risk and switching intention to CC is positively moderated by personal innovativeness.
H6: The relationship between reward sensitivity and switching intention to CC is positively moderated by personal innovativeness.
H7: The relationship between knowledge and switching intention to CC is positively moderated by personal innovativeness.

**Fig 1:**
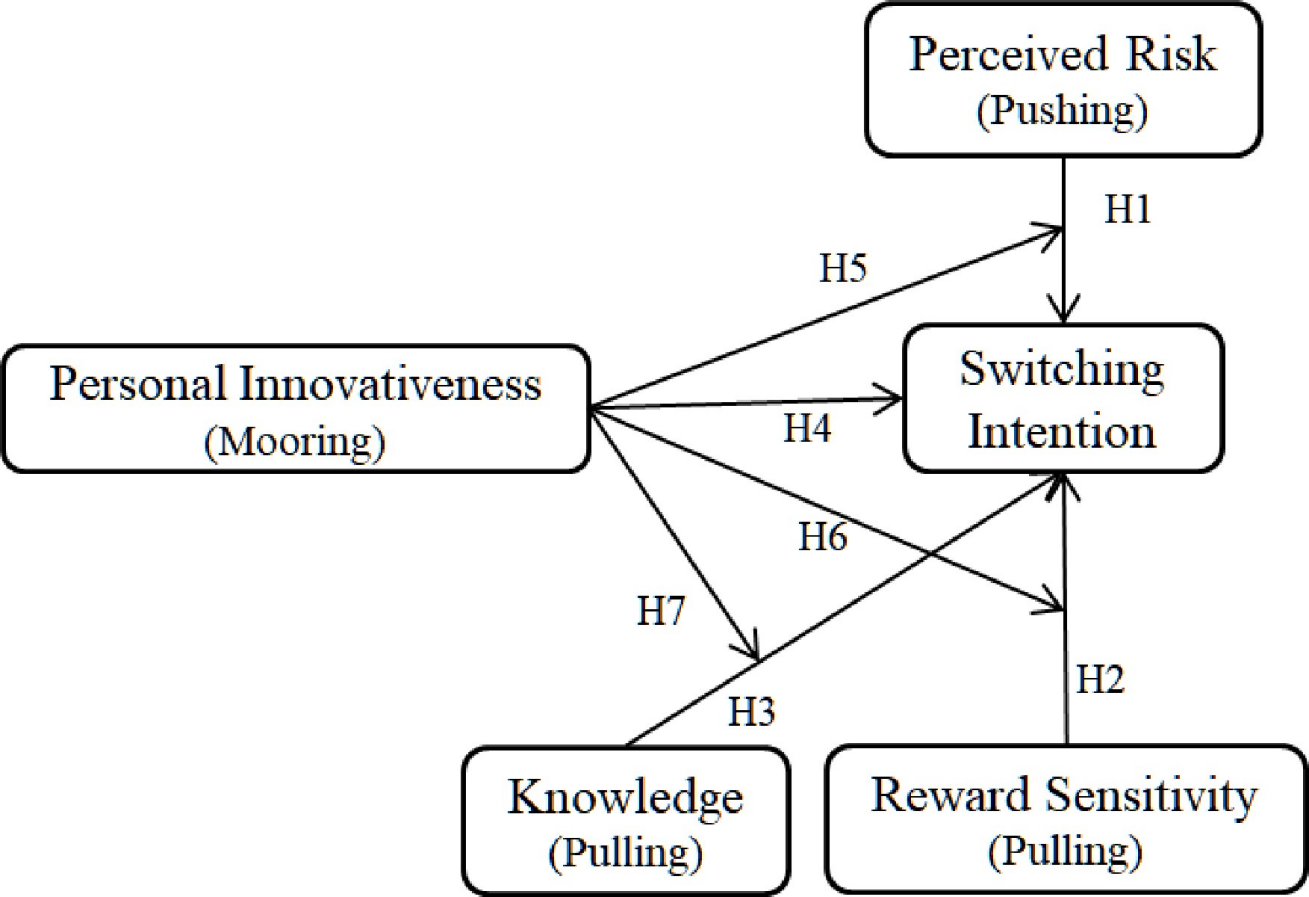
Proposed research model.

The proposed model is showed in Fig 1.

## 3. Measurement and data analysis

It was necessary to survey individual investors that have investment experience in CC and are also familiar with it. All participants should agree a disclaimer before taking part in the survey. The disclaimer is 1) I volunteered to take part in this anonymous survey. 2) All information is authorized to be public. 3) No conflict of interest exists. Therefore, all participants are voluntarily and anonymously to join in this survey. We collected data from members of several investment clubs in South Korea and China. The reasons are explained as follows: 1) Most of them are middle class and private bank customers of multinational financial institutions who also invest by themselves. 2) They share CC investment experience and useful information in social network groups and regularly participate in investment parties offered by financial institutions. 3) Because all of the individual investors have their own business in different field, the deviation of sampling scope is avoided. 4) All the individual investors have business with those financial institutions for years, which proves that they have launched substantial investment works. 5) Shareholders of those financial institutions are from different countries, which ensure the neutrality and randomness of sampling respondents. This study also included a pretest (*N* = 45) to confirm the validity and reliability of the final survey. The pretest respondents were asked to give advice to improve the quality of the survey overall, and the final survey questionnaire was updated according to their suggestions [47]. With help of peers of those financial institutions, we delivered online questionnaires among individual investors who have invested in CC. The questionnaires were distributed among 332 people from January 20 to February 9, 2019. In total, 275 respondents returned questionnaires, giving response rate of 83%. Excluding questionnaires incompletely or carelessly answered, 244 responded questionnaires were used for data analysis.

The perceived risk construct was measured using three items. (“It is inconvenient to find good investment target in traditional financial market.” “It is not wise to spend a lot of time to invest in traditional financial market.” “It costs too much to get satisfying expected return on traditional financial market.”). A 7-point Likert scale test was applied in the survey

The reward sensitivity construct was measured using three items. (“A good opportunity to get profits from CC can motivate me to invest in it.” “In most cases, I prefer to do something that pays off soon.” “I want to be the best of people around me”). A 7-point Likert scale test was applied in the survey.

The knowledge construct was measured using three items. (“I am informed about what CC can offer.” “I am knowledgeable about CC.” “I am aware of CC”). A 7-point Likert scale test was applied in the survey.

The personal innovativeness construct was measured using three items. (“I am always the first to try it out among my colleagues and peers.” “Overall, I’d like to try and experiment with new things.” and “Interesting and high return investment projects always make me look for ways to experiment with them”). A 7-point Likert scale test was applied in the survey.

The switching intention construct was measured using three items. (“I am likely to switch to invest in CC.” “I desire to switch to invest in CC.” “I plan to switch to invest in CC.”). A 7-point Likert scale test was applied in the survey.

### 3.1 Methodology and data analysis

The structural equation model (SEM) is a statistical method for analyzing the relationship between variables based on the covariance matrix of variables. It is an important tool for multivariate data analysis. It is a combination of multiple statistical analysis methods that can simultaneously test factors, analysis items and relationships between errors. PLS (partial least squares) analysis is an appropriate method to be applied in the related analysis of this study because it can easily test the moderating effects in a relatively small data samples. Compared with other SEM tools, PLS-SEM has some advantages, such as small sample size and applications with little available theory. Therefore, PLS has been used in many different research fields including social network service, e-commerce, business administration and investment [47]. PLS-SEM analysis also includes varieties of estimations such as reliability, validity, R square, path estimates and t-values to assess our research model as well as measurements. The Ethics Committee of Economic School of Anyang Normal University waived the need for ethical approval.

### 3.2 Descriptive statistics

Among the 244 participants, 161 were men (65.9%) and 83 were women (34.1%). Most of them are well educated professionals with high income. 91% of them have more than 3 years’ investment experience which identifies them to be mature personal investors. 47.5% of the respondents are relatively young individual investors who are between 30 and 40 years old. Table 2 shows the respondents of demographics.

**Table 2:** Demographic statistics.

Category	Subject	N	%
Gender	Male	161	65.9%
	Female	83	34.1%
Education Level	High School	33	13.5%
	Bachelor	105	43.0%
	Master	82	33.6%
	Ph.D.	24	9.9%
Age	20–30	62	25.4%
	31–40	116	47.5%
	41–50	53	21.7%
	More than 50	13	5.4%
Yearly Income	<$ 50,000	78	31.9%
	≥$50,000&<$100,000	109	44.7%
	≥$100,000&<$200,000	47	19.3%
	≥$200,000	10	4.1%
Term of Investment Experience	<3 years	22	9.0%
	≥3 & <5 years	96	39.3%
	≥5 & <10 years	101	41.4%
	≥10 years	25	10.3%
Main investment Amount	<$100,000	69	28.2%
	≥$100,000 & <$300,000	138	56.6%
	≥$300,000	37	15.2%
Occupation	Finance	53	21.7%
	IT	85	34.8%
	Service Industry	89	36.5%
	Others	17	7.0%

## 4. Results

When composite reliability’s value is above 0.70, the reliable measures will be consistent in their values and stable and free from error in the multiple measurements. Cronbach’s α means a questionnaire is reliable when its value is above 0.70. AVE reflects how much of the variation explained by each latent variable comes from all the items in the latent variable. When the AVE value is above 0.50, it indicates that the latent variable has good convergence validity. When Standard factor loading’s value is above 0.70, it confirms that item can represent the connotation of the construct. The analysis progress is standard and results are consistent with previous researches [48,49,50]. Table 3 shows the Cronbach’s alpha values, and all of them are above the recommended threshold value of 0.7 to prove their reliability. Composite reliabilities are also above the recommended threshold value of 0.7 to prove their convergent validity. AVEs are above the recommended threshold value of 0.5 to prove their convergent validity.

**Table 3:** Convergent validity, composite reliabilities testing results.

Construct	Item	Standardized Factor Loading	AVE	Composite Reliability	Cronbach’s α
Perceived Risk	PR1	0.911			
	PR2	0.827	0.854	0.903	0.927
	PR3	0.815			
Reward Sensitivity	RS1	0.845			
	RS2	0.854	0.810	0.866	0.913
	RS3	0.834			
Knowledge	KN1	0.883			
	KN2	0.941	0.812	0.892	0.908
	KN3	0.817			
Personal Innovativeness	PI1	0.885			
	PI2	0.867	0.902	0.910	0.913
	PI3	0.857			
Switching Intention	SI1	0.829			
	SI2	0.961	0.821	0.857	0.917
	SI3	0.851			

Discriminant validity is used to test if a construct is distinct with each other. It is examined by comparing the square roots of average variance extracted with the coefficient of correlations between constructs. When the positive square roots of average variance extracted values are greater than the coefficient of correlations between constructs, discriminant validity exists. The analysis progress is standard and results are consistent with previous researches [51,52,53].Table 4 shows that AVE’s square root is higher than other values related to other constructs’ correlations. Table 5 indicates that the items’ self-factor loadings are higher than other items’. The two steps have proved the model’s discriminant validity. The two tests together prove the discriminant validity exists in this research. Therefore, all related tests have proved that the model is reliable and valid for evaluating the structural model.

**Table 4:** Descriptive statistics, correlation matrix and square roots of AVE.

Construct	Mean	SD	PR	RS	KN	PI	SI
PR	4.011	1.174	0.924				
RS	4.114	1.542	0.221	0.901			
KN	4.009	1.872	0.354	0.454	0.901		
PI	3.874	1.158	0.377	0.645	0.422	0.949	
SI	4.445	1.875	0.157	0.757	0.554	0.447	0.907

**Table 5:** Loadings and cross-loadings.

	PR	RS	KN	PI	SI
PR1	**0.715**	0.454	0.565	0.289	0.588
PR2	**0.724**	0.522	0.523	0.454	0.547
PR3	**0.731**	0.431	0.541	0.454	0.456
RS1	0.428	**0.841**	0.514	0.445	0.564
RS2	0.218	**0.824**	0.447	0.354	0.665
RS3	0.257	**0.774**	0.487	0.266	0.456
KN1	0.481	0.644	**0.754**	0.347	0.545
KN2	0.533	0.454	**0.852**	0.245	0.654
KN3	0.361	0.508	**0.814**	0.265	0.254
PI1	0.457	0.512	0.254	**0.747**	0.654
PI2	0.281	0.245	0.551	**0.755**	0.455
PI3	0.698	0.454	0.607	**0.868**	0.421
SI1	0.530	0.355	0.421	0.565	**0.908**
SI2	0.424	0.456	0.428	0.498	**0.857**
SI3	0.475	0.356	0.426	0.374	**0.814**

**Fig 2:**
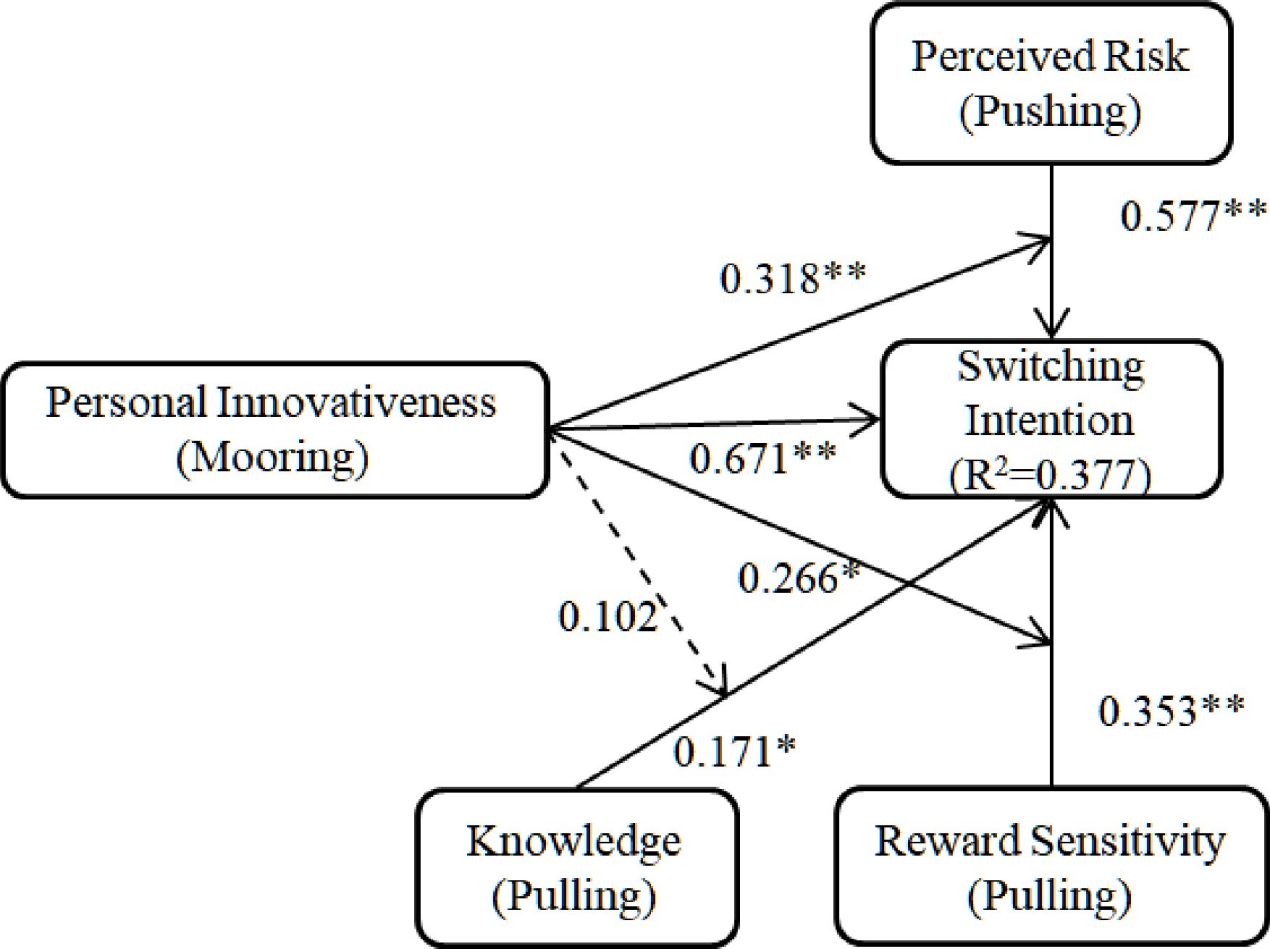
Structural model. standardized coefficients. ** p < 0.01; *0.05≥p≥0.01.

Path analysis for the research model predicting on the whole data is shown in Table 6. Fig 2 shows standardized path coefficients and corresponding t-values. In conjunction with the degrees of freedom, t-value is used to calculate the corresponding significance of p value. The larger t-value is, the smaller the corresponding p value is. (0.05≥p≥0.01) is considered statistically significant, indicated by a single (*). And (0.01>p) is considered to be highly statistically significant, indicated by (**). In Table 5, t-statistics confirmed that all paths except one were highly significant. The analysis progress is standard and results are consistent with previous researches [54–57]. Personal innovativeness, perceived risk knowledge and reward sensitivity proves to have positive effect on switching intention to CC. Personal innovativeness proves to have positive moderating effects on relationships between perceived risk reward sensitivity and switching intention, and proves to have no moderating relationship between knowledge and switching intention. Specially, perceived risk (0.577) and personal innovativeness (0.671) had very strong impacts on switching intention, which can be assumed that perceived risk and personal innovativeness are relatively more important factors affecting switching intention than reward sensitivity and knowledge. Fig 2 indicates PLS analysis results, by testing the estimates and R^2^, whereas Table 5 shows details of hypotheses testing results of estimates and t-values.

**Table 6:** Hypotheses testing results of estimates and t-values.

Hypotheses	Path	Estimate	t-value	Result
H1	Perceived Risk(PR)	0.577	7.421**	Accepted
	→ Switching Intention			
H2	Reward Sensitivity (RS)	0.353	3.854**	Accepted
	→ Switching Intention			
H3	Knowledge (KN)	0.171	2.195*	Accepted
	→ Switching Intention			
H4	Personal Innovativeness (PI)	0.671	8.693**	Accepted
	→Switching Intention			
H5	PI moderating	0.318	3.157**	Accepted
	PR and SI			
H6	PI moderating	0.266	2.446*	Accepted
	RS and SI			
H7	PI moderating	0.102	0.842	Not Accepted
	KN and SI			

## 5. Discussion and contribution

Drawing on the PPM and RST theories, the proposed model is used for explaining perceived risk, personal innovativeness, reward sensitivity and knowledge’s direct influence on switching behavior, as well as the moderating influences of personal innovativeness on the relationship between switching intention and its antecedents. Taking into consideration the importance of crypto-currency related factor in affecting individual investors’ switching intention behavior, this research investigates on how individual investors integrate crypto-currency into their asset portfolio to increase return of investment.

The research’s empirical evidence indicates that the main drivers of individual investors’ switching intention are, in the order of impact, personal innovativeness, perceived risk, reward sensitivity and knowledge. Interestingly, personal innovativeness plays the most important role in the research model. The reason could be due to that most of the individual investors of CC are well educated middle class with plenty of investment experience, which makes them ambitious to attempt to new investment objects for better return. Especially, knowledge is not as significant as the other factors. The explanation could be that the trading platforms have offered operation interfaces similar to traditional stock market and conducted plenty of online and offline education and trainings for potential customers because popularity of crypto-currencies has increased a lot in recent years and more and more multiple tools and information resources have appeared. Moreover, perceived risk shows to have significant affection on switching intention. It may be due to that most traditional financial investment markets players are institutional investors, and it is impossible for individual investors to compete with institutional investors in capital size, information consulting resources and information analysis ability. For CC investment field, since only limited large institutional investors have participated in, the perceived risk among individual investors may be less than traditional financial investment markets.

This study contributes to the current research in several perspectives. Firstly, since only limited previous researches apply information system theories into CC financial investment science, the most pioneer work of this study is to integrate two popular theories (PPM & RST) of information system to investigate roles of crypto-currencies in investment behavior field. Specially, because seldom of previous empirical researches in crypto-currency have ever use SEM-PLS in explaining factors affecting crypto-currency’s investment switching intention, this study tries to introduce SEM-PLS in this field to make a fresh start in exploring behavior financial investment science.

Additionally, this study firstly combines behavior finance science with information management science to investigate the individual investors’ adoption of crypto-currency, which provides empirical evidence to fill the gap. Meanwhile, the results indicate that most individual investors have more than 3 years’ investment experience in traditional financial markets and it’s impossible for them to abandon the traditional financial markets. As a matter of fact, the two markets can compete and develop in parallel.

## 6. Conclusions and limitations

In this study we introduced the basic concept of PPM theory and empirically studied factors affecting individual investors’ switching intention to CC. It is expected to see a steady growth of CC investment, however, CC investment among individual investors is still in the early stage and, therefore, there are many issues to be resolved before widespread CC investment can be adopted by individual investors. The major finding of the present study suggests that individual investor would like to adopt hybrid structures when confronted with attractive options or new investment opportunities [34]. The results show that perceived risk, personal innovativeness, reward sensitivity and knowledge are key issues among individual investors. Personal innovativeness proves to have positive and effective moderating effect on relationships between perceived risk, reward sensitivity and switching intention. The reason that personal innovativeness doesn’t play a moderating role on switching and knowledge could be that individual investors who possess more innovativeness are more intent to try on CC investment rather than to collect plenty of relevant knowledge about CC to avoid losing investment opportunities.

Drawing on RST and push-pull-mooring theory, this research proves that the individual investors are not only attracted by significant expected return from CC but also relevant knowledge and risks disclosed by CC market regulators and distributors. Thus, CC markets regulators and distributors are responsible to carry out marketing strategy to improve individual investors’ approval and recognition. Additionally, reducing handling fees, updating transaction data in real time and providing the fastest way of cash arrival will be warmly received by individual investors in improving their expected return and reward. Moreover, it is meaningful to use ceaseless conduct propaganda to spread knowledge among individual investors to increase access to information for individual investors.

From the individual investors’ point of view, building a proper financial portfolio including unrelated assets will significantly reduce investment risks. Since many regulators around the world have already ruled on the risks involved in investing in crypto assets, publishing studies on their own websites and other sites as well as regulated the different activities of the crypto-currency sector, it is wise for individual investors to pay attention to those rules and improve their knowledge about distinguishing different CCs’ characteristics in order to find the most suitable ones to construct their own portfolios that could bring expected returns or rewards. Furthermore, individual investors can join the online or offline investment clubs to look for like-minded investors to share ideas and get rational advices in the process of investment to improve their own innovation ability. Moreover, although CC is not safe as a single investment object due to its dramatic price fluctuation in a short time, CC asset can be used as tools of risk hedging, speculation and long-term holding and play an important role in individual investors’ portfolios. Individual investors can combine CC asset with other assets which have negative correlations to achieve better expected return. Additionally, CC can also be applied in much more areas by individual investor, such as cross-border investment.

Although this research has tried to give a thorough exploration on CC investment field, there are some limitations that can’t be ignored. The first limitation lies in the short consistency of the research results of different area contexts, which should be tested and verified in future studies. Also, future studies may consider different brands of CC’s characteristics and situational factors. Furthermore, sincere there are more and more nations have decided to have their own CC, it may be necessary to compare the characters based on different nations’ economic strategies.

## Supporting information

S1 Data (CSV)

S2 Data Switching intention to cryptocurrency market: Factors predisposing some individuals to risky investment (20-01-2019). (DOCX)
