# Supplementary material for: Switching intention to crypto-currency market: Factors predisposing some individuals to risky investment
Source: PLoS One. 2020 Jun 4;15(6):e0234155. doi: 10.1371/journal.pone.0234155 (PMC7272014; doi:10.1371/journal.pone.0234155)
Supplement: S2 Data — (DOCX) [file pone.0234155.s002.docx]

**Switching Intention to Cryptocurrency Market: Factors predisposing Some Individuals to Risky Investment** (20-01-2019)

If you agree, please paste√in the bar of “I agree”

| **Disclaimer** | |
| --- | --- |
| 1. I volunteered to take part in this anonymous survey.  2. All information is authorized to be public.  3. No conflict of interest exists. | I agree______ |

Please paste√ in the bar of your choice

|  | Male | Female |
| --- | --- | --- |
| Gender |  |  |

Please paste√ in the bar of your choice

|  | High School | Bachelor | Master | Ph.D |
| --- | --- | --- | --- | --- |
| Education level |  |  |  |  |

Please paste√ in the bar of your choice

|  | 23-30 | 31-40 | 41-50 | More than 50 |
| --- | --- | --- | --- | --- |
| Age |  |  |  |  |

Please paste√ in the bar of your choice

|  | <50,000 | ≥50,000&<100,000 | ≥100,000&<200,000 | ≥200,000 |
| --- | --- | --- | --- | --- |
| Yearly Income  (us dollar) |  |  |  |  |

Please paste√ in the bar of your choice

|  | <3 years | ≥3&<5 years | ≥5&<10years | ≥10years |
| --- | --- | --- | --- | --- |
| Term of Working Experience |  |  |  |  |

Please paste√ in the bar of your choice

|  | <100,000$ | ≥100,000&<300,000$ | ≥300,000$ |
| --- | --- | --- | --- |
| Main investment Amount |  |  |  |

Please paste√ in the bar of your choice

|  | Finance | IT | Service Industry | Others |
| --- | --- | --- | --- | --- |
| Occupation |  |  |  |  |

Please paste√ in the bar of your choice (1-strongly disagree; 7-strongly agree)

| perceived risk | ① | ② | ③ | ④ | ⑤ | ⑥ | ⑦ |
| --- | --- | --- | --- | --- | --- | --- | --- |
| It is inconvenient to find good investment target in traditional financial market. |  |  |  |  |  |  |  |
| It is not wise to spend a lot of time to invest in traditional financial market. |  |  |  |  |  |  |  |
| It costs too much to get satisfying expected return on traditional financial market. |  |  |  |  |  |  |  |

Please paste√ in the bar of your choice (1-strongly disagree; 7-strongly agree)

| reward sensitivity | **①** | **②** | **③** | **④** | **⑤** | **⑥** | **⑦** |
| --- | --- | --- | --- | --- | --- | --- | --- |
| A good opportunity to get profits from CC can motivate me to invest in it. |  |  |  |  |  |  |  |
| In most cases, I prefer to do something that pays off soon. |  |  |  |  |  |  |  |
| I want to be the best of people around me |  |  |  |  |  |  |  |

Please paste√in the bar of your choice (1-strongly disagree; 7-strongly agree)

| knowledge | **①** | **②** | **③** | **④** | **⑤** | **⑥** | **⑦** |
| --- | --- | --- | --- | --- | --- | --- | --- |
| I am informed about what CC can offer. |  |  |  |  |  |  |  |
| I am knowledgeable about CC. |  |  |  |  |  |  |  |
| I am aware of CC. |  |  |  |  |  |  |  |

Please paste√in the bar of your choice (1-strongly disagree; 7-strongly agree)

| personal innovativeness | **①** | **②** | **③** | **④** | **⑤** | **⑥** | **⑦** |
| --- | --- | --- | --- | --- | --- | --- | --- |
| I am always the first to try it out among my colleagues and peers. |  |  |  |  |  |  |  |
| Overall, I’d like to try and experiment with new things. |  |  |  |  |  |  |  |
| Interesting and high return investment projects always make me look for ways to experiment with them. |  |  |  |  |  |  |  |

Please paste√in the bar of your choice (1-strongly disagree; 7-strongly agree)

| switching intention | **①** | **②** | **③** | **④** | **⑤** | **⑥** | **⑦** |
| --- | --- | --- | --- | --- | --- | --- | --- |
| I am likely to switch to invest in CC. |  |  |  |  |  |  |  |
| I desire to switch to invest in CC. |  |  |  |  |  |  |  |
| I plan to switch to invest in CC. |  |  |  |  |  |  |  |
